# Enhanced magnetic-field-induced optical properties of nanostructured magnetic fluids by doping nematic liquid crystals

**DOI:** 10.1186/1556-276X-7-249

**Published:** 2012-05-15

**Authors:** Xiang Wang, Shengli Pu, Hongzhu Ji, Guojun Yu

**Affiliations:** 1 College of Science, University of Shanghai for Science and Technology, Shanghai, 200093, China

**Keywords:** Magnetic fluids, Nematic liquid crystals, Ferronematic materials, Birefringence, Figure of merit of optical properties

## Abstract

Ferronematic materials composed of 4-cyano-4′-pentylbiphenyl nematic liquid crystal and oil-based Fe_3_O_4_ magnetic fluid were prepared using ultrasonic agitation. The birefringence (Δ*n*) and figure of merit of optical properties (*Q* = Δ*n*/α, where α is the extinction coefficient) of pure magnetic fluids and the as-prepared ferronematic materials were examined and compared. The figure of merit of optical properties weighs the birefringence and extinction of the materials and is more appropriate to evaluate their optical properties. Similar magnetic-field- and magnetic-particle-concentration-dependent properties of birefringence and figure of merit of optical properties were obtained for the pure magnetic fluids and the ferronematic materials. For the ferronematic materials, the values of *Q* increase with the volume fractions of nematic liquid crystal under certain fixed field strength and are larger than those of their corresponding pure magnetic fluids at high field region. In addition, the enhancement of *Q* value increases monotonously with the magnetic field and becomes remarkable when the applied magnetic field is beyond 50 mT. The maximum relative enhanced value of *Q*_R_ exceeds 6.8% in our experiments. The results of this work may conduce to extend the pragmatic applications of nanostructured magnetic fluids in optical field.

## Background

Magnetic fluid (MF) is a kind of stable colloidal suspension of small magnetic particles with typical sizes of 10 nm. The liquid carrier may be either an aqueous or a nonpolar solvent. The nanoparticles are coated with a surfactant layer, such as oleic acid and polymer, which will prevent agglomeration by overcoming the van der Waals attractive forces between the particles. The Brownian motion can keep the nanoparticles from settling under gravity [1]. MF was firstly invented in the mid-1960s, and then the study and applications of MF have been extended to multidisciplinary sciences, such as chemistry, fluid mechanics, and magnetics. Recently, with the fast development of MF, it has been broadly used for dynamic sealing, shock absorbers, audio loudspeaker coolant, and biomedical sciences [2,3]. Because of the dramatic development of optical communication and integrated optics, the optical properties of MF have attracted a great deal of attention from researchers since the late part of the twentieth century, which include tunable refractive index [4], birefringence [5], Faraday effect [6], optical transmittance [7,8], optical scattering [9,10], and so on. Most of these are based on the fluid behavior and magnetism of the MF. Until now, several potential applications of MF to optical devices have been proposed, such as MF optical switches [11], MF gratings [12], MF light modulation [13], MF optical fiber modulator [14], and MF optical limiting [15]. Recently, some experimental investigations about the magneto-optical effects of binary, multiple-phase, ionic, and doped MFs show that these kinds of MFs can present some unique optical properties [16-19].

Nematic liquid crystal has attracted a great deal of attention from researchers owing to its optical birefringence and scattering properties, which can be controlled by the external stimuli, such as electrical, magnetic fields, and shear stresses [20]. Usually, a strong magnetic field is needed to study the magnetic-field-induced optical properties of liquid crystals. This is due to the small anisotropic diamagnetic susceptibility (Δ*χ*) of the liquid crystal [21]. To lower the applied magnetic field, liquid crystal doped with ferromagnetic grains (the corresponding mixture is denoted as ferronematic material) has been proposed [21,22]. Burylov and Raikher have reported that magneto-optical response of a liquid crystalline system can be enhanced by uniformly doping with ferromagnetic grains and the orientational state of the ferronematic material can be fully controlled by a rather weak magnetic field (much less than 10 mT) [23]. Raikher and Stepanov have discussed a transient birefringence response of a nematic liquid crystal doped with single-domain ferromagnetic particles and have derived a set of coupled macroscopic equations describing the evolution of the director texture and the magnetization distribution within the ferronematic material during the change of the external field [24]. It has been disclosed that many properties of liquid crystal can be improved and enhanced by doping with ferromagnetic grains, which are favorable for practical applications [25-28].

Considering the unique properties of MFs and ferronematic materials, doping MFs with nematic liquid crystal is attractive and needs further in-depth investigation. To the best of our knowledge, few experiments about the magnetic-field-induced birefringence Δ*n* and figure of merit of optical properties *Q* (*Q* = Δ*n*/α, where α is the extinction coefficient) of this composite have been done. This work will experimentally investigate the magnetic-field- and concentration-dependent Δ*n* and *Q* of the as-prepared ferronematic materials. The results of this work may be helpful to better understand the optical properties of nematic liquid crystal-doped MFs (ferronematic materials) and extend the applications of MFs in optical field.

## Methods

### Preparation of ferronematic materials

The ferronematic materials investigated in this work are a mixture of MFs and nematic liquid crystals. The oil-based Fe_3_O_4_ MF with volume fraction of 5.62% was provided by Ferrotec Corporation (Chuo-ku, Tokyo, Japan). The 4-cyano-4′-pentylbiphenyl (C_18_H_19_N) nematic liquid crystal (5CB) was provided by Shijiazhuang Huarui Scientific and Technological Co., Ltd (Shijiazhuang, Hebei, China). The MF was diluted with the liquid paraffin carrier (provided by Sinopharm Chemical Reagent Co., Ltd, Shanghai, China) with ultrasonic agitation of about 2 h. The as-prepared pure MF samples have four different concentrations of magnetic particles, referred as samples a (1:8), b (1:10), c (1:12), and d (1:14). The numbers in the brackets indicate the volume ratio of MF to liquid paraffin used to mix. The volume fractions of the magnetic particle for samples a, b, c, and d are 0.62%, 0.51%, 0.43%, and 0.37%, respectively, as shown in Table 1.

**Table 1:** Pure MF samples with different concentrations of magnetic particles

**Sample**	**Volume ratio of pure MF to liquid paraffin**	**Volume fraction of magnetic particle**
a	1:8	0.62%
b	1:10	0.51%
c	1:12	0.43%
d	1:14	0.37%

To investigate the optical properties of ferronematic materials with different concentrations of magnetic particles, 1 ml of 5CB and 3 ml of any one of sample a, b, c, or d are mixed to obtain samples a-5CB, b-5CB, c-5CB and d-5CB, respectively. The volume fractions of 5CB of these samples are the same (25.00%). Their magnetic particle volume fractions are 0.47%, 0.38%, 0.34% and 0.28%, respectively, as shown in Table 2.

**Table 2:** Concentrations of samples a-5CB, b-5CB, c-5CB, and d-5CB

**Sample**	**Volume ratio of sample a, b, c, or d to 5CB**	**Volume fraction of magnetic particle**	**Volume fraction of 5CB**
a-5CB	3:1	0.47%	25.00%
b-5CB	3:1	0.38%	25.00%
c-5CB	3:1	0.34%	25.00%
d-5CB	3:1	0.28%	25.00%

Similarly, a series of samples with a constant concentration of magnetic particle and different concentrations of 5CB are prepared and shown in Table 3. They are denoted as samples MF-5CB, MF-5CB(2), MF-5CB(3), and MF-5CB(4). The volume fraction of magnetic particle is fixed at 0.50%. Their volume fractions of 5CB are 23.08%, 19.34%, 15.24%, and 10.72%, respectively.

**Table 3:** Concentrations of samples MF-5CB, MF-5CB(2), MF-5CB(3), and MF-5CB(4)

** Sample**	**Volume fraction of magnetic particle**	**Volume fraction of 5CB**
MF-5CB	0.50%	23.08%
MF-5CB(2)	0.50%	19.34%
MF-5CB(3)	0.50%	15.24%
MF-5CB(4)	0.50%	10.72%

### Experiments

**Figure 1:**
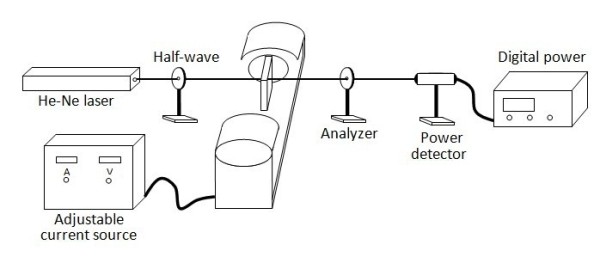
Schematic of the experimental setup for measuring the birefringence of the ferronematic materials.

The magnetic-field-induced birefringence of the as-prepared ferronematic samples can be measured by the light extinction method, which consists of a sample cell placed between accurately set ‘crossed’ polarizers as shown in Figure 1[29]. A single-mode He-Ne laser emitting linearly polarized light with a wavelength of 632.8 nm is employed. The propagation direction of the incident light is normal to the applied magnetic field. The thin film sample is placed between a pair of solenoids, which generate a uniform horizontal magnetic field in the sample region. The strength of magnetic field can be adjusted by tuning the magnitude of the supply current. For the birefringence measurement, the optimum angle of the polarization direction of the incident linearly polarized light with respect to the magnetic field should be around π/4 [30]. At a given magnetic field, the light transmittance after the analyzer is investigated. Through rotating the analyzer, the maximum and minimum transmitted intensities (*I*_max_ and *I*_min_) are measured, respectively. The birefringence Δ*n* can be determined according the following:

(1) $$\Delta n\text{=arcsin}\frac{2\sqrt{I_{\min}/I_{\max}}ch\left(h_{1}-h_{2}\right)}{1+I_{\min}/I_{\max}}\lambda /\left(2\pi d\right)\text{,}$$

where *d* equals 170 μm and *λ* is 632.8 nm; $h_{i}\left(i=1,2\right)$ are the electric field absorption coefficients for the polarization direction of the incident light paralleling and being perpendicular to the magnetic field, respectively, which can be obtained by solving $I_{i}=I_{\mathrm{oi}}e^{-2h_{i}\left(H\right)}$_._$I_{\mathrm{oi}}$ and $I_{i}$ are the transmitted intensities of the sample under zero and certain magnetic field. Therefore, the birefringence of the sample as a function of magnetic field can be acquired by measuring *I*_max_, *I*_min_, *I*_*oi*_, and *I*_*i*_ at different magnetic fields.

## Results and discussion

**Figure 2:**
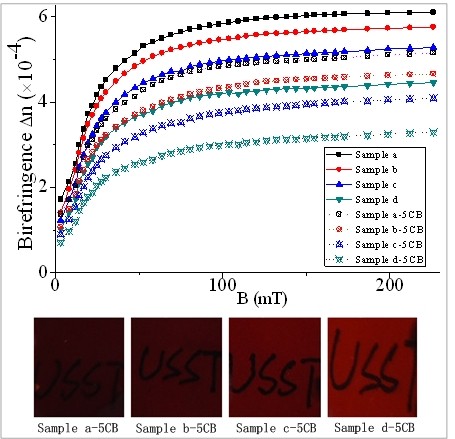
**Birefringence of pure MFs and MFs doped with 5CB.** Birefringence of pure MFs with different magnetic particle concentrations and MFs doped with 5CB as a function of magnetic induction.

Figure 2 shows the birefringence of pure MFs with different magnetic particle concentrations as a function of magnetic induction. From Figure 2, we can see that the birefringence of samples a to d increases gradually with the magnetic induction and tends to saturate at high field. This is attributed to the formation of magnetic chains under external magnetic field. Application of external magnetic field will induce structural ordering within the MF confined between two glass plates, which are due to the interaction between the magnetic dipoles induced at the particle sites [31,32]. This results in the optical anisotropy of MF under applied magnetic field. When the applied magnetic field is small, the aligned discrete magnetic short chains will form. With further increasing the strength of the magnetic field, the short chains will start to combine with each other to become longer discrete chains. The longer the chain structure is, the stronger the spatial anisotropy is and the larger the birefringence of MF becomes.

Figure 2 also indicates that samples with high volume fraction of magnetic particle have high birefringence under the same field strength. Low concentration of magnetic particles will lead to weak interaction between magnetic particles under the fixed field. The average distance between the magnetic particles within the samples will then become relatively large. Therefore, the number of chains per unit area will decrease as the concentration of magnetic particles decreases, which contributes to the low degree of structure anisotropy and then small value of birefringence of the sample under low field.

The experimental results for the corresponding ferronematic samples (samples a-5CB, b-5CB, c-5CB, and d-5CB) are also shown in Figure 2, which are very similar to those of the pure MF samples. Table 2 shows that all the ferronematic samples have the same volume fraction of 5CB (25.00%) but have different magnetic particle volume fractions, so the variation in birefringence between different ferronematic samples is attributed to the change of magnetic particle concentration. Besides, the birefringence of the ferronematic samples is weaker than those of the corresponding pure MF samples. This is probably assigned to the decrease of magnetic particle concentration through doping 5CB.

**Figure 3:**
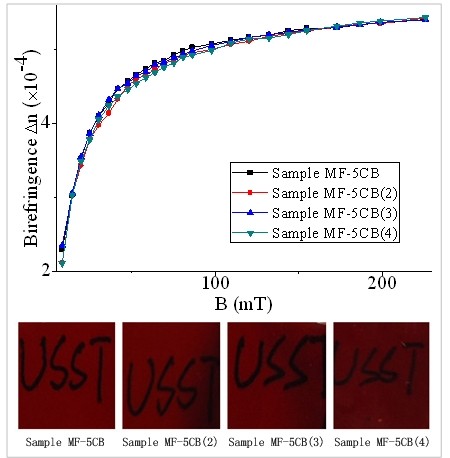
**Birefringence of samples with different volume fractions of 5CB as a function of magnetic induction.** The magnetic particle volume fraction is fixed.

To further investigate the influence of 5CB concentration on the birefringence of ferronematic samples, the birefringence of samples MF-5CB, MF-5CB(2), MF-5CB(3), and MF-5CB(4) is also measured, and the experimental results are shown in Figure 3. Table 3 shows that all the ferronematic samples have the same volume fraction of magnetic particles (0.50%) but have different 5CB volume fractions. The Δ*n*-B curves for different samples in Figure 3 almost overlap with each other, which mean that only the magnetic particle concentration is crucial to the birefringence of the samples, and 5CB concentration has no influence on the birefringence of the samples.

**Figure 4:**
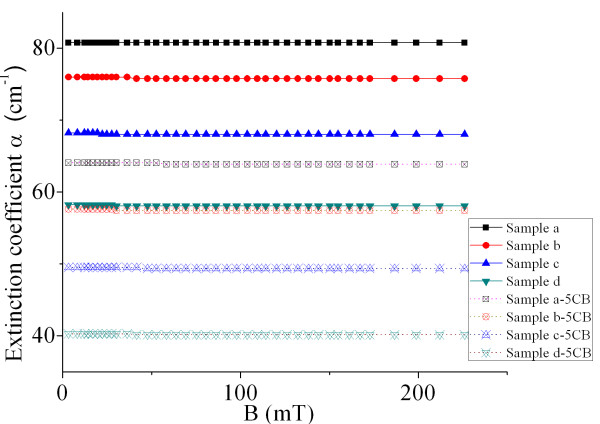
**Extinction coefficients of pure MFs and doped MFs.** Extinction coefficients of pure MFs with different magnetic particle concentrations and MFs doped with 5CB as functions of magnetic induction.

For most practical applications to MF-based photonic devices, the values of birefringence and transmittance of the materials are two very critical parameters. Pure MFs and ferronematic materials with higher magnetic particle concentration have higher absorption, though they have a higher value of birefringence. The figure of merit of optical properties *Q* defined as Δ*n*/α may be appropriate to evaluate their optical properties. The larger the value of *Q* is, the better the optical properties of the samples are. Though 5CB does not contribute to the birefringence, it will lessen the extinction coefficient of the sample, which will be beneficial to enhance the value of *Q*. To obtain the value of *Q*, the extinction coefficient α of the first two series of samples (Tables 1 and 2) as a function of magnetic induction are measured and shown in Figure 4. Figure 4 indicates that the extinction coefficient does not change with the field strength for a given sample. Moreover, the sample with higher volume fraction of magnetic particle has a larger extinction coefficient.

**Figure 5:**
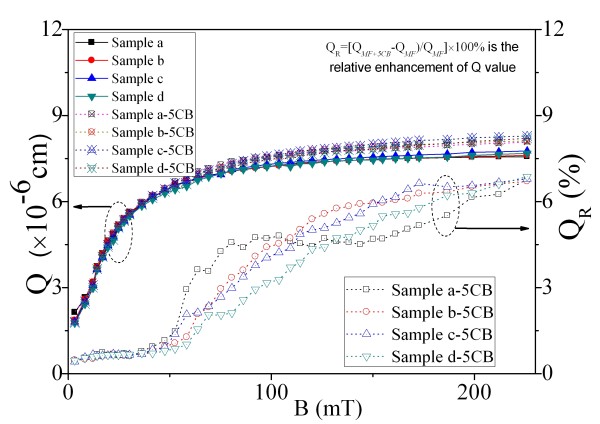
**Figure of merit of optical properties and the relative enhancement of the*Q*value.** Figure of merit of optical properties of the pure MFs and MFs doped with 5CB and the relative enhancement of Q value of the corresponding ferronematic samples as functions of magnetic induction.

The calculated value of *Q* of the pure MFs and their corresponding ferronematic samples as a function of magnetic induction is plotted in Figure 5. From Figure 5, we can see that the value of *Q* for samples a to d increases gradually with the applied magnetic field and tends to saturate at high field, while the value of *Q* does not vary with the magnetic particle concentration under the same field strength. This means that the magnetic particle concentration has no influence on the figure of merit of optical properties of the samples. The magnetic-field-dependent values of *Q* for the corresponding ferronematic samples (samples a-5CB, b-5CB, c-5CB, and d-5CB) are very similar to those of the pure MF samples. However, the ferronematic samples have larger values of *Q* than their corresponding pure MF samples at high field region. When doped with 5CB, the magnetic particle concentration of the ferronematic samples will reduce compared with the corresponding pure MF samples. This will result in the decrease of birefringence and the increase of transparency of the sample. The experimental results in Figure 5 indicate that the latter effect outweighs the former one at high field region. This leads to the augment of the value of *Q*, i.e., enhanced optical properties for the ferronematic materials at high field region. Figure 5 also implies that 5CB is crucial to the figure of merit of optical properties of the samples and that magnetic particle concentration has no influence on the figure of merit of optical properties of the samples.

To quantify the enhanced optical properties, the relative enhancement of the *Q* value defined as $Q_{\text{R}}=\left[\left(Q_{\text{MF}+5\text{CB}}-Q_{\text{MF}}\right)/Q_{\text{MF}}\right]\times 100$ as a function of magnetic field is calculated and plotted in Figure 5. Herein, *Q*_MF_ and *Q*_MF+5CB_ are the *Q* values at a certain magnetic field for the pure MF samples and their corresponding ferronematic material samples, respectively. Figure 5 shows that *Q*_R_ increases with the applied magnetic field and tends to saturate at high field. When the externally magnetic field *B* is less than 50 mT, the *Q*_R_ is slight, while *Q*_R_ becomes notable when *B* is larger than 50 mT. Comparing with the *Q* value of the pure MF samples, the relative enhancement of *Q* value of the corresponding ferronematic samples (*Q*_R_) can reach about 6.8%.

**Figure 6:**
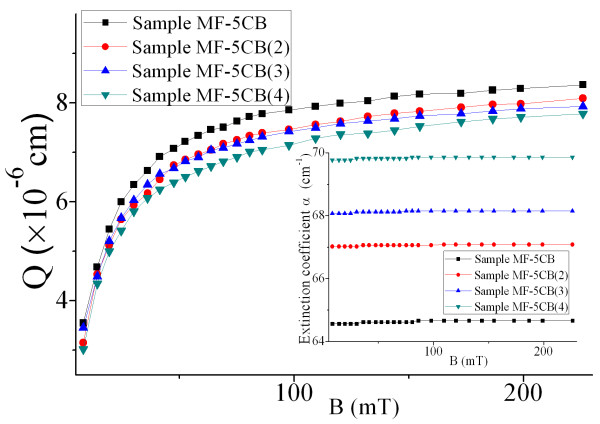
**Extinction coefficient and figure of merit of samples as functions of magnetic induction.** With fixed magnetic particle volume fraction and different volume fractions of 5CB.

To investigate the influence of 5CB concentration on the extinction coefficient and figure of merit of optical properties of the ferronematic samples, experiments are done with samples MF-5CB, MF-5CB(2), MF-5CB(3), and MF-5CB(4), and the results are shown in Figure 6. Figure 6 shows that the extinction coefficient does not change with the field strength for a given sample and that the ferronematic sample with higher volume fraction of 5CB has a smaller extinction coefficient.

Figure 6 also indicates that the value of *Q* increases gradually with the applied magnetic field and tends to saturate at high field. Moreover, the higher the volume fraction of 5CB is, the higher the value of *Q* is under certain fixed field strength. Therefore, the value of *Q* for the ferronematic materials can be tuned by adjusting the volume fraction of 5CB.

## Conclusions

The optical properties (birefringence and figure of merit of optical properties) of the pure MF and ferronematic thin films under externally applied magnetic field are investigated. The pure MF and the ferronematic samples show the similar magnetic-field-dependent properties of birefringence and figure of merit of optical properties, which increase with magnetic induction and tend to saturate at high field. Both types of samples with high volume fraction of magnetic particle have high birefringence. Besides, the ferronematic material with high volume fraction of 5CB has a relatively high value of *Q* under a certain fixed field strength. The experimental results reveal that the magnetic particle concentration is crucial to the birefringence of the samples and that 5CB concentration has no influence on the birefringence of the samples. However, the 5CB concentration is crucial to the figure of merit of optical properties of the ferronematic samples, and magnetic particle concentration has no influence on the figure of merit of optical properties of the ferronematic samples. The *Q* values of the ferronematic materials are larger than those of their counterparts (pure MFs). The maximum relative increase in *Q* value is around 6.8% for our experimental samples.

## Abbreviations

5CB: 4-cyano-4′-pentylbiphenyl (C_18_H_19_N) nematic liquid crystal; MF: Magnetic fluid.

## Competing interests

The authors declare that they have no competing interests.

## Authors' contributions

The experiment measurement and data calculation of this study were mainly done by the first author, XW. SP contributed to both the theoretical and experimental work and gave some useful suggestions to XW. GY and HJ contributed to experimental work and gave XW some help. This article was written by XW and revised by SP. All authors read and approved the final manuscript.

## Authors' information

SP is an associate professor at the College of Science, University of Shanghai for Science and Technology. His research interests focus on advanced photonic materials and magneto-optics. XW, HJ, and GY are pursuing their master's degrees.
